# ‘Motivational work’: a qualitative study of preventive health dialogues in general practice

**DOI:** 10.1186/s12875-020-01249-z

**Published:** 2020-09-08

**Authors:** Marie Broholm-Jørgensen, Siff Monrad Langkilde, Tine Tjørnhøj-Thomsen, Pia Vivian Pedersen

**Affiliations:** 1 National Institute of Public Health, Research Program on Health and Social Conditions, University of Southern Denmark, Studiestræde 6, 1455 Copenhagen K, Denmark; 2 The Danish Centre for Urban Regeneration and Community Development, Hvidovre, Denmark

**Keywords:** Denmark, General practice, Prevention, Preventive health dialogues, Motivation, qualitative research

## Abstract

**Background:**

The aim of this article is to explore preventive health dialogues in general practice in the context of a pilot study of a Danish primary preventive intervention ‘TOF’ (a Danish acronym for ‘Early Detection and Prevention’) carried out in 2016. The intervention consisted of 1) a stratification of patients into one of four groups, 2) a digital support system for both general practitioners and patients, 3) an individual digital health profile for each patient, and 4) targeted preventive services in either general practice or a municipal health center.

**Methods:**

The empirical material in this study was obtained through 10 observations of preventive health dialogues conducted in general practices and 18 semi-structured interviews with patients and general practitioners. We used the concept of ‘motivational work’ as an analytical lens for understanding preventive health dialogues in general practice from the perspectives of both general practitioners and patients.

**Results:**

While the health dialogues in TOF sought to reveal patients’ motivations, understandings, and priorities related to health behavior, we find that the dialogues were treatment-oriented and structured around biomedical facts, numeric standards, and risk factor guidance. Overall, we find that numeric standards and quantification of motivation lessens the dialogue and interaction between General Practitioner and patient and that contextual factors relating to the intervention framework, such as a digital support system, the general practitioners’ perceptions of their professional position as well as the patients’ understanding of prevention —in an interplay—diminished the motivational work carried out in the health dialogues.

**Conclusion:**

The findings show that the influence of different kinds of context adds to the complexity of prevention in the clinical encounter which help to explain why motivational work is difficult in general practice.

## Background

While treatment plays an important role within general practice, prevention of so-called lifestyle-related diseases has become an urgent issue in health politics as well as an important task in general practice [1]. The current Danish Government’s health policy understands prevention as reducing recurrence of illness and prevention of chronic conditions [2]. This understanding also frames prevention in the Danish general practice. However, it has been emphasized that general practitioners (GP) lack time and resources to focus on primary prevention, and that the increased focus on individual health behavior counselling affects the GP-patient relationship by placing strain on trust in the relationship [3–6]. Worrying that they might harm trust in the GP-patient relationship, GPs have been found to be reluctant to address lifestyle-related issues [4, 7, 8]. Additionally, preventive care in general practice has reported a lack of patient motivation and willingness to change health behaviors [9, 10] as well as low compliance with GPs’ recommendations [6], which could prompt skepticism among the GPs concerning the impact of preventive initiatives in general practice [7]. Existing research reveals that the daily primary care practice, dominated by diagnoses, treatment, and secondary prevention, is an additional barrier to the implementation of primary preventive care in general practice [6, 11]. Further research is needed to understand how prevention is carried out in general practice. The aim of this article is, therefore, to provide in-depth insight into the unfolding of preventive health dialogues in general practice from perspectives of both GPs and patients. The empirical material is based on a pilot study of a Danish primary preventive intervention, named ‘TOF’ (a Danish acronym for ‘Early Detection and Prevention’). The aim of the TOF intervention is to identify individuals at high risk of lifestyle-related disease (health conditions that are predominantly caused by health-risk behaviors, such as poor diet, smoking, high consumption of alcohol, or lack of exercise) and to provide targeted and coherent preventive services in the primary healthcare sector, including general practice [12]. Analytically, we focus on is the clinical encounter between GPs and patients in the context of preventive health dialogues.

### The concept of motivational work

KL Frohlich, E Corin and L Potvin [13] propose viewing health behaviors, such as participating and acting upon preventive health dialogues, as social practices that are reinforced by and emerge in relation to other people and the possibilities of action presented by the social context. This understanding contrasts with the bio-medical perspective of behavioral change as individual attributes estranged from the context that frames peoples’ lives [13]. Drawing upon Frohlich et al.’s perspective, we analyze health behavior as an interpersonal affair, which occurs differently in different social contexts and situations. Motivation to change health behavior is from this perspective neither a stable condition nor a priori given but instead a process and a ongoing work carried out in an interplay between individuals and people around. To understand this interplay—and the social complexities it implies in general practice—we developed the concept ‘motivational work’ from a dialogue between theoretical concepts and the empirical material and used it as an analytical lens. Similarly to the Canadian-American psychologist Albert Bandura, we regard self-efficacy as an important influence on people’s motivation and behavior [14]. However, in this study we additionally pay attention to the various outside factors that influence the social meeting between GPs and patients and thus the possibilities for action in the health dialogues. Hence, motivational work refers to the compound of actions and words employed to facilitate motivation for behavioral changes in the context of preventive health dialogues. As a consequence, we regard health dialogues as social meetings that include negotiations of identity, authority, knowledge and values [11, 15]. We thus understand motivational work as an interplay between GPs and patients, which is influenced by contextual circumstances such as the differing risk perceptions and expectations of both GPs and patients as well as the physical and organizational conditions framing the clinical encounter. By introducing motivational work, we wish to direct attention to the social encounter between GPs and patients as well as to the interactions that arise in preventive health dialogues in the context of TOF.

## Methods

### Design

With this study, we strive for transparency and reflexivity in the data collection. Hence, in the following sections we demonstrate the steps made in the process from data collection to interpretation, hereby accounting for the different aspects of validity [16]. We approached the preventive health dialogues with different methods and informants, which both nuanced and shed light on the issues at stake. The article draws upon fieldwork and qualitative interviews from a process evaluation of health dialogues in general practice carried out in 2016 in relation to the TOF pilot study. As the process evaluation was inspired by the realistic evaluation method [17], this rendered possible an analytical focus on the working mechanisms in the health dialogue and the conditions for implementation, e.g. GPs’ and patients’ attitudes, competencies, collaborations, and interactions. Through observations of health dialogues and semi-structured interviews with both GPs and patients, we obtained a multifaceted view on the health dialogues. By combining observations and semi-structured interviews, we gained insight into the interaction between GPs and patients and how they experienced the health dialogue. Thereby, we obtained insight into different dimensions of the empirical object [18].

### Setting: The ‘Early Detection and Prevention’ intervention (TOF)

The TOF intervention, that was tested in the pilot study, systematically identifies individuals at high risk of lifestyle-related disease (health conditions that are predominantly caused by health-risk behaviors, such as poor diet, smoking, high consumption of alcohol, or lack of exercise) and provides targeted and coherent preventive services in the primary healthcare sector, including general practice [12].

Early detection and the provision of coherent preventive services are expected to diminish the individual and societal burden of chronic disease [12]. To achieve this, the TOF pilot study consisted of 1) a stratification into one of four groups based on results from an online questionnaire to patients (see Table 1), 2) a digital support system for both GPs and patients, 3) an individual digital health profile for each patient, and 4) targeted preventive services in either general practice or a municipal health center. In this article, we focus on those patients who were at high risk of developing lifestyle-related diseases and thus eligible for the offer of a focused health examination from and a health dialogue with the GP (Group Two).

**Table 1:** Risk stratification in TOF pilot study

Group	Definition
1	Participants with a pre-existing diagnosis and/or in current treatment for a lifestyle-related disease
2	Participants at high risk of developing lifestyle-related disease and thus eligible for the offer of a targeted intervention at the GP
3	Participants engaging in health-risk behavior and thus eligible for the offer of a targeted intervention at the municipality
4	Participants with a healthy lifestyle and no need for further intervention.

The health dialogues between GPs and patients lasted approximately 30 min and were based on the results from the health examination, the remarks in the digital health profile, and patient’s answers to questions in the questionnaires [12]. During the health dialogues, GPs were required to base the dialogue on a number of topics provided by the digital support system to ensure that all adverse health behaviors identified in the questionnaire and health examination were considered and addressed. The health dialogue consultations were based on the 5A model (Assess, Advice, Agree, Assist, Arrange follow-up) [19] and elements of motivational interviewing (MI), including methods of goal-setting, scaling questions and action-planning [20, 21]. MI was recommended to be followed in order to uncover patients’ motivation, wishes, understandings and priorities in terms of their own health in a patient-centered dialogue [22]. In this line of thinking, the GPs’ role in the TOF pilot study was to 1) motivate and advise patients to change health-risk behavior without taking over responsibility for their actions, 2) to help patients clarify their prospective health-risk behavioral changes, and 3) to enter goal-setting and action-planning in the digital support system during the dialogues [12]. Although the health dialogues were based on the elements of MI, the GPs did not receive any training in using MI prior to the intervention.

### Recruitment of study participants

All study participants—GPs and patients—were recruited from the TOF pilot study. Prior to the commencement of the pilot study, all enrolled GPs were invited to a three-hour training course, which among other things included a presentation of the process evaluation [12]. The GPs were then contacted by e-mail and/or phone after the study commencement for recruitment to participate in observations and interviews. The GPs furthermore provided information to SML about which health dialogues could be observed.

### Collecting empirical material

The empirical material consists of 10 observations of preventive health dialogues between GPs and patients, followed by seven semi-structured interviews with GPs and 11 semi-structured interviews with patients who participated in health dialogues. Because the process evaluation focused on the preventive measures in TOF, such as the 5A model, we observed only the health dialogues and not the health examinations. The interviews, though, also included informants’ experiences with the health examinations.

SML conducted all observations and interviews. Prior to each observation and interview, GPs were asked permission before SML contacted the patients by phone to receive verbal consent about the qualitative study.

Before each observation, SML greeted the patient in the waiting room to create confidence, deepened the purpose of the observation, and clarified the role as an observant (as passive observer not being an active part of the health dialogue). The observations took place within the GP’s consultation room during the health dialogue, where SML was placed on a chair behind the patient. SML took fieldnotes during the health dialogue and expanded the notes into a coherent text on the same day. As the health dialogues lasted 30 min each, approximately 5 hours of observations were undertaken. Immediately after the health dialogues, SML carried out interviews with the patients either at the patient’s home, in a consultation room at the GP, or at a nearby café. Six female and five male patients aged between 40 and 60 years participated in the study. Due to traffic delay, one of the interviewed patients was not observed during the health dialogue. Inspired by a realistic evaluation approach, the interviews were structured around the themes of prevention and concerned patients’ perspectives on the health dialogue, the applicability of the digital support as well as their perception of, motivation and ability to participate in the TOF pilot study (see Additional file 1 for the full set of questions for the patients in the study). The interviews lasted 30 to 60 min. Given that patients in this study participated on the grounds of a risk stratification, attention was paid to avoid generating concerns regarding health and risk of illness and patients’ responses to risk classifications in the interviews [18, 23]. SML achieved this by means of a flexible interview guide that allowed an openness to the issues that patients deemed central. Furthermore, the fact that SML was an external evaluator and did not have a health professional background allowed for neutrality in the interview situation.

Within 2 days after the observed health dialogues, SML conducted interviews with the GPs, either at the GPs’ office or by telephone. In the interviews, GPs were asked to elaborate upon their experiences with and perceptions of the health dialogues, the applicability of the digital support, and their overall assessment of the TOF pilot study (see Additional file 2 for the full set of questions for the GPs in the study). The interviews lasted up to 30 min. All informants were informed of the ethical principles involved regarding confidentiality and anonymity, including that any quotes would be assigned pseudonyms. All signed an informed consent form for participation in the interview or observation study.

All interviews were recorded on a digital audio recorder and were transcribed verbatim. All quotes in this article has in anonymous form been translated from Danish to English by a professional translator.

### Analysis

The empirical material was analyzed using both an open coding and a thematic coding to uncover general observations, regularities, convergences, and divergences derived from the material [24]. With a focus on actions and words employed to motivate behavioral changes, the authors thoroughly read the interview transcripts and observation notes. Analytical themes were grouped into categories, and during a continuous analytical process—moving between the empirical material and theoretical concepts, such as risk perspectives and management—categories were confirmed or modified. Being open to ambiguities and complexities in perceptions and actions regarding health and focusing on interactions in the context of the health dialogue, ‘motivational work’ came up as an analytical lens that guided our attention to the factors influencing how GPs assessed and addressed patients’ motivation to change adverse health behavior. To ensure validity selected excerpts, preliminary results, and interpretations were presented and discussed with selected experts and research colleagues.

## Results

Our results are structured around three themes. First, we show how motivation was practiced during the preventive health dialogues. Second, we illustrate how patients perceived the preventive focus on health behavior in general practice. Third, we focus on how both GPs and patients had certain expectations regarding the role of the GP, which affected the motivational work carried out during the health dialogues.

### Practicing motivational work

We introduce the results with an ethnographic observation that shows a significant empirical example of how motivational work most typically was practiced in the preventive health dialogue in the TOF intervention. As we shall argue, motivational work in the health dialogues occurred as one-way communication, in which the GP controlled, instead of facilitated, interplay and dialogue with the patient.

The doctor looks at the screen and confirms that the diet is red (in the risk zone). He asks Kenn whether the health survey has got him thinking about anything. Kenn answers that, yeah, he knows. “Know what?” asks the doctor? Kenn explains that he knows he is overweight and eats too much. He eagerly explains how he’s currently experimenting with leaving candy out all the time to wean himself off eating the whole thing in one go. He talks about an experiment he saw on TV in which a kindergarten kept candy out all the time and only brought out carrots every so often, with the result that the children didn’t want to eat candy but instead rushed over to the carrots. The doctor listens. Kenn continues explaining how he had previously almost been underweight, but after he had stopped smoking, he had gained a bit more weight, a bit more, a bit more. The doctor does not comment on this but glances quickly around Kenn’s health profile and comments instead on his responses regarding smoking and alcohol. He returns to diet shortly thereafter. He asks whether Kenn is familiar with BMI – Body Mass Index – and says: “It should ideally be a maximum of 25 – yours is at 27. What about exercise?” Kenn says that it could be better and explains that he does gymnastics once a week and goes hunting during the season. The doctor asks whether it’s sort of classic men’s gymnastics, “The kind of thing where you don’t sweat?” he asks with a laugh in his voice. Kenn smiles and assures him that they really work hard. The doctor smiles, “Joking aside.” He looks at Kenn: “It’s important to get your heartrate up and break a sweat. Do you have a bike?” he asks. “No, I just hate biking!” Kenn answers. The doctor does not respond but turns around in his chair and looks at the computer screen. “Then I need to ask you. In terms of increasing your efforts in terms of physical activity, would you maybe be interested in a service from the municipality?” Kenn looks a bit skeptical. “What is it?” he asks. The doctor explains that the municipality has a lifestyle service for adults with overweight and a place where you can learn about health. Kenn still looks skeptical. “Hmm… Let’s assume that I’m interested. I mean, that I’m looking for somewhere I can learn about obesity. As a smoker, I didn’t need it.” The doctor looks at the description of the municipality’s service and reads it aloud. A moment later, Kenn acquiesces: “Alright, let’s accept it … Then we’ll have done something at least. It’s not necessarily certain that it would happen here, internally.” The doctor fills out some boxes in the health profile. He points at the screen, where an image shows a scale of 1 to 10. “How motivated are you in terms of the municipality’s lifestyle team?” he asks. Kenn thinks. “It’s probably a 4 or a 5…” “OK,” answers the doctor, types in the number and clicks around on the screen. He prints out a description of the municipality’s service. While the printer is going, he asks, “So, how’s the strategy with the bowl of candy going?” Kenn smiles. “Surprisingly well!” he answers. “The total amount at least has gone down – also when it comes to heavy food.” The doctor responds, “Great!” and hands the information on the municipality’s service over to Kenn in printed form. “If you’re really hardcore, there’s also a nutritionist. It isn’t free, but then you’re setting the agenda.” Kenn does not seem particularly interested as the doctor writes the nutritionist’s contact details down for him. The doctor clicks further in the health profile and asks Kenn a couple of quick questions about medicine use, etc. Afterwards, the doctor talks about cholesterol totals, and he calculates Kenn’s 10-year risk and a current risk with the help of a special computer program. “Hey, it looks really good – 1% risk.” Kenn smiles. “OK, then, it really can’t be much better.” The doctor responds, “It looks really good overall – ideally, you should lose a few kilos! … I also think you need to work on getting motivated to give it a shot – I believe in you! … But should we say that’s that, then? Then you’ll continue the program with the municipality.” (GP 2, Patient 9)

In this example, the GP attempted to motivate to behavior change in different ways. The health dialogue started out with the doctor asking an open-ended question that caused the patient to account for his candy experiment. The patient thereby presented the GP with an opening regarding his motivation to change eating behaviors. Although the dialogue started with including the patient’ experience, the GP did not follow up on this information, but instead applied an action perspective: recommending the patient to start biking and try consulting a nutritionist, which resulted in a drop in the patient’s motivation. Overall, throughout the empirical material, we found motivational work to be characterized by guidance, which included information, suggestions, and advice on risk factors, such as: “It’s important to get your heart rate up and break a sweat. Do you have a bike?” (GP 2, Patient 9), “You should lose weight to lower your cholesterol level,” (GP 3, Patient 4) or “Try to get 30 minutes of exercise every day, get the heart rate up… go for a daily walk” (GP 6, Patient 8). Although these examples are not only biomedically anchored, but also include ways of approaching patients everyday lives, the dialogues in general seldom included the patients’ experiences with behavioral changes, general life situation, or social circumstances directly, which could influence their motivation, ambivalence, and actions regarding health behavior.

Furthermore, the GPs reduced MI tools, such as scaling questions and goal setting, to a means of gaining numerical information instead of serving as a dialogue tool. The numeric standards and measurements constituted preventive work on which the GPs never followed up. That is, the GPs neither asked questions about the reason for the assessment nor questions about the patient’s specific motivations for changing behavior. As such, perspectives and experiences that the GPs could have utilized to activate and strengthen the patient’s motivation for change [25] remained empty information, and information about behavior was quantified and given in generic terms by the GP, isolated from any social influences [13, 26]. One explanation for the GPs approach to motivation could be the framework and the instructions of the TOF-intervention, which the GPs were to follow during the health dialogues. The following example from an observation of a health dialogue shows how the GP acted to meet the expectations of the intervention.

GP 1: “You are going on a smoking cessation course – now I’m just putting words in your mouth (laughs)”. *The sound of the printer printing the offer.* “Let’s agree on a goal. Let’s agree that you go from 10 to five cigarettes a day. We will write that you will stop smoking within a year – then they will be happy in there [TOF project group] …Complete smoking cessation we will write”. Patient 1 does not reply. GP 1 reads out loud:” How sure are you that you will reach your goal?”. GP 1 looks patient 1 in the eyes. Patient 1: “Unsure”. GP 1 reads out loud: “Does the patient understand and accept?”. GP 1 looks at patient 1. Patient 1: “….yes yes…[Laughs and shakes his head]. (GP 1, patient 1)

The performance of motivation in this study could be explained by the structure of the digital framework, which facilitated the GPs using the digital support system as a checklist rather than as a starting point of a dialogue. Furthermore, in the interviews with the patients, we found that when GPs did not follow the digital support system, patients felt that their stories and individual experiences were seen and heard. In this way, the digital support system, which was meant to ensure that all the patients’ identified adverse health behaviors were considered and addressed, diminished the GPs’ use of the elements of MI. Thus, we argue that the framework and the instructions of the intervention influenced the character of the interaction between GP and patient and in this way the motivational work.

We further argue that the approach to MI presented in the examples can be characterized as a treatment-oriented practice rooted in a biomedical perspective, characterized by a numeric objectification of bodily functions and symptoms based on classification systems for diagnosing somatic and mental diseases, one that less so include patients’ experiences, values, or everyday lives [27, 28]. Existing research shows that GPs’ focus on diagnoses and treatment affects whether prevention is introduced in the clinical encounter [6, 29]. These findings correlate well with the findings in this article. However, the clinical encounter is more complex than the GPs only thinking in biomedical terms and patients only about their everyday lives. As we will show in the next section, patients also adhere to the biomedical rationale.

### Experiencing motivational work

In this section, we illustrate how patients experienced and perceived the preventive focus on health behavior in general practice and how this influenced the motivational work carried out in the health dialogues.

Generally, the patients stated that they had not previously considered seeking advice or guidance from their GP about lifestyle related issues. Several patients did not regard risk factors, such as obesity, as a disease and, as a result, they did not present it as a health problem to the GP. The ways in which patients separated lifestyle related issues from disease are reflected in the following interview extract:

“When I contact the doctor, it’s because I notice something particular. If there’s something unusual, or there’s something that’s changed, and I notice it, then I get in touch with the doctor. But not my lifestyle, no … Because I feel I’ve got that covered” (Patient 7).

According to a study by BP Mjølstad, AL Kirkengen, L Getz and I Hetlevik [30], the contents of conversations between GPs and patients are framed by an awareness of what is not appropriate to share with the GP, such as everyday life issues. These authors argue that this awareness is socially and culturally embedded in the Western society, where patients are taught to regard the body in a physical and biomolecular manner. This social embedment lessens the degree to which patients introduce experiences from their everyday lives in the assessment of symptoms in the encounter [30]. In our study, patients understood health behavior as a private matter and therefore not an appropriate content for the conversations with the GP:

“If it’s about how I have to alter my lifestyle, then it’s really more on the home front where that kind of thing happens. I mean, if we’re sitting around the table and agree, well, ‘we’d better eat a bit more salad and more beet burgers,’ then that’s where it’s decided. Not with the doctor. It’s nice of her to try, but no” (Patient 6).

As illustrated by this quote, patients generally regarded lifestyle related issues as something they themselves were responsible for changing and as something that took place at home and not in the GP’s consultation room. Patients’ awareness of their health behavior was in this way also often connected to a biomedical understanding of health behavior as an individual affair, as opposed to other clinical health issues. Instead of agreeing to the preventive premise of the health dialogue and of the intervention guidelines, patients often brought other health problems into the conversation, for example eczema, tennis elbow, and birthmarks. Other studies additionally find that patients coproduce the implementation of lifestyle promotion in an active or passive way [5], by for instance anticipating how the GPs might assess their health behavior and by incorporating this in the dialogue [25]. Which could help explain why patients shifted the focus and reproduced a treatment-oriented focus in the preventive health dialogues in our study. In a study examining shame and honor in the clinical encounter, shifting focus in preventive consultations has been identified as a means by which patients preserve or regain face when confronted with behavior that is deemed insufficient [15]. Though based on the patients’ understandings of the clinical encounter and health behavior as presented above, we found that by shifting focus, the patients contributed to the health dialogue as one-way information provided by the GP, with a focus on objectified biomedical, classifications of bodily functions and symptoms.

### Expectations of GPs’ role in prevention

In this section, we demonstrate how both GPs’ and patients’ expectations of the GPs’ role affected the motivational work carried out in the health dialogues.

Generally, the GPs expressed concern about becoming overbearing and scaring patients away when confronting them with advice about health behavioral change. Some GPs expressed skepticism about setting goals in the health dialogues and about patients’ willingness to change health behavior. One GP described an awareness of not “pursuing” the patients:

“[In terms of setting goals,] well… they talk it up a bit while they’re sitting here, and when they get home, then they forget about it? I mean, if they say: ‘Yeah, but it’s a 7.’ Then I don’t know whether they’re going to follow up on it. But I mean, I don’t want to force them into it either. I don’t want to pursue them. I don’t want to punish them. Nah. I have one with these really bad feet, and she can’t have surgery unless she quits smoking. […] So, I say, ‘But, well, can’t you quit smoking?’ She basically can’t do that – what’s she supposed to do? She can’t walk, she can’t smoke. So, I mean… (laughs) it’s hard. […] But you can say to them: ‘Well, I mean, that’s just how it is.’ – and then, well, we don’t need to talk about it anymore (GP 1).

Focusing the health dialogue on biomedical facts and treatment has in other studies been found to stem from GPs’ practical inability to carry out MI as well as lack of time and concerns about harming trust in the doctor-patient relationship [4, 8, 11, 31]. GPs have been found to balance authority and respect for patients’ autonomy by compromising on or sidestepping certain health issues to avoid harming their relationships with patients, which has consequences for prevention in the clinical encounter [4, 32]. This means that GPs’ professional commitment to treatment, professional authority, and respect for patients’ autonomy may dominate the motivational work and dialogue with patients in the health dialogues. This was also evident in our empirical material.

Additionally, the excerpt above illustrates that after conveying normative biomedical facts to patients, such as the risks of smoking, motivational work was understood to be completed. This implies a hidden assumption that what the GP says is in itself a motivational factor. The term “the doctor-drug” [33] is widely known and describes how the GP’s mere presence influences patients’ responses to illness and treatment. As such, GPs’ perception of their professional authority affects the social practices of motivation for health behavioral change that emerges from and is reinforced within the context of the clinical encounter [13]. This correlates well with the findings in this study, as the GPs’ comprehension of their professional position and focus on individual-oriented treatment seemed to influence the ways in which GPs understood and performed their role in the preventive health dialogues. However, contextual circumstances, such as the framework of the pilot study, may have presented a barrier to the GPs’ inclusion of the patients’ perspectives. For example, use of the digital support system in the health dialogues was new to the GPs, which may have influenced the degree to which the GPs had the resources to focus on the patients’ perspectives. An increase in time spent looking at the computer screen has in other studies been found to affect patient-GP interaction by leading to more periods of silence and a decrease in dialogue and information sharing [34, 35]. As a result, and as shown previously, the digital support system functions as a checklist, which may cause GPs to read questions aloud and to enter patients’ answers without making use of the information in active interplay with the patients.

Nonetheless, it is worth noting that the patients in this study expected GPs to focus on medical treatment and not on health behavior and prevention.

“No one said anything about this being a lifestyle change project – because then I don’t actually think I’d have agreed to take part, because that’s not something I need. I thought it was supposed to be about my health” (Patient 6).

Patients perceived the GP as someone who treated illness and as someone who could attend to health problems that they could not handle on their own. We found that patients perceived issues related to health behavior, such as obesity and smoking, as self-inflicted, self-controlled, and not (yet) disease related. As a result, the contexts – the patients’ private lives versus the biomedical context framing the clinical encounter – affected whether the patients perceived and recognized health behavior as an appropriate health problem. Patients’ attention to not burden or waste GPs’ time [30, 36] may explain why patients anticipated and assessed health behavior as an individual affair.

The following example illustrates the divergence between the processual motivation to change health behavior on the one hand and the biomedical, rational treatment focus on the other hand.

“I know that I shouldn’t smoke, and I know that it’s not healthy, and I know all that. But I’m just not ready for it, right? I’ve tried quitting many times – and it just hasn’t worked yet. And I just think, ‘Well, but, as long as I keep it to under 10 cigarettes a day and am conscious of not increasing it, then I’ll probably decide on my own whether to quit smoking in a year, in three months, or whenever I do. … But the doctor, well, he wants me to set a date. And there, I just thought to myself, ‘I’ll be damned if he’s deciding that’” (Patient 1).

Quantifying and estimating the length of this process by pushing the patient to set a date for smoking cessation disregarded the patient’s previous experiences with attempting this, which resulted in a decline in motivation. A qualitative study of health behavior counselling in general practice demonstrated that although GPs and practice nurses showed awareness of the value of including patients in the preventive health dialogue, the provision of simple risk factor information was the predominant strategy [37]. Our findings reveal that ensuring a patient-centered dialogue and enhancing intrinsic motivation for behavioral change was complicated partly by GPs quantification of prevention and providing risk factor advice isolated from the patients’ social context and partly by patients’ understandings and expectations of appropriate health problems to discuss with the GP. K Thomas, P Bendtsen and B Krevers [5] suggest that prevalent understandings of the implementation of health behavioral change in healthcare could be improved if patients were seen as co-producers rather than receivers. Findings in the present study, however, suggest that this would not shift focus away from information about health behavior in generic terms, given that the patients additionally demonstrated expectations about the content and structure of the health dialogue based on a biomedical rationale.

To summarize, we found that GPs’ and patients’ expectations regarding the structure and content of the health dialogue influenced the character of the motivational work. Our findings show that both GPs and patients in an interplay— influenced and reduced MI in the health dialogues to one-way information due to a treatment-oriented focus and expectations related to perceptions of prevention as an individual and private task.

## Concluding discussion

This article provides important insight into the complexity of preventive health dialogues between GPs and patients in general practice. By introducing the concept of ‘motivational work’, we found that both GPs and patients performed a treatment-oriented focus in the health dialogues. While the health dialogues in theory sought to reveal patients’ motivations, desires, understandings, and priorities related to health behavior [12], we show that, in practice, the dialogues were treatment-oriented and structured around biomedical facts, numeric standards, and risk factor guidance.

An inherent assumption of the TOF intervention is that early detection and coherent prevention services can reduce the risk of chronic disease [12]. In the TOF pilot study, prevention was systematically provided through an invitation to a health and risk assessment. This means that the assessment could point to health-related problems that patients may not have recognized as problematic. However, motivation for health behavioral change has generally been found to occur when patients experience symptoms affecting their quality of life and affected by conditions surrounding their everyday lives and to a lesser extent influenced by information about risk [10, 38–41]. Furthermore, although the TOF pilot study provided the patients with a risk profile and offered a digital health profile, which they were encouraged to look through to prepare for the health dialogue, we found that the risk profiles did not affect the health dialogues as such. Thus, we have not included this information in the study. Though, one reason for the risk profiles being absent could be that patients had not ascribed this information as problematic in their everyday lives. Therefore, giving information about risks that are neither perceived nor affect patients’ lives directly seems counterproductive as the general feeling of health is given more value [41].

Overall, we find that that numeric standards and quantification of motivation lessens the dialogue and interaction between GP and patient and eliminates the focus on the patient’s general life situation. While motivation in MI is understood as the reason for people’s actions, desires, needs, and direction to behavior, and as an important prerequisite for lifestyle changes [21], we found that combining the elements of MI with the digital support system meant that the GPs in the TOF intervention needed to consider contradictive approaches to prevention in the health dialogues. Instead of serving as a dialogue tool to uncover patients’ motivation, wishes, understandings and priorities, we found that the digital support system quantified information about health behavior in generic terms. A review that examined the use of health information technology such as electronic health records, eye contact, information sharing, building relationship and pauses in the conversation and how these things impacted in the clinical encounter, indicates that the computer, and thus the use of a digital support system in the health dialogues, influences both communication between GPs and patients and the possibilities for actions [34]. While the findings in our study showed that patients perspectives were included when GPs did not follow the digital support system rigorously, we argue that the digital support system provided by TOF hindered the participating GPs use of the MI elements. Following the line of thought of realistic evaluation, the framework of TOF changed the conditions of preventive consultations in general practice when introducing the digital support system, and thus altered the conditions of motivational work in the health dialogues [17]. Based on this unintended consequence of the intervention, we suggest that the digital support system should prospectively act as a guidance to generate motivation and not as a checklist. Although this permits a higher degree of flexibility and thus, complexity to the intervention [42], we argue that this could increase the GPs’ possibilities to clarify patients’ motivations in terms of their own health. Furthermore, we argue that the participating GPs should have a thorough introduction to of the digital support system to ensure identification of and focus on patients’ prospective health-risk behavioral changes. With these findings, we do not argue against conducting interventions such as the TOF intervention, but instead propose including the wider context of intervention to facilitate the possibility of understanding why things are done within the contexts that frame the social action. This can bring forth important information about relevant content for and implementation of an intervention.

In this article, we illustrate that patients held expectations of what was appropriate to discuss in the clinical encounter with the GP. This is not a unique finding [30, 36]. Consequently, public health interventions that operate solely at the GP level may risk falling short as targeted individuals tend not to be susceptible to prevention in this context. FA Derksen, TC Olde Hartman, JM Bensing and AL Lagro-Janssen [43] propose that longer consultations could influence whether attention is paid to prevention in the clinical encounter. Our findings, however, do not support this suggestion, given that patients in the health dialogues brought up other medical health problems despite the intended focus on prevention. Qualitative studies of complex preventive interventions have shown that participants ascribe different expectations to their participation than assumed in the intervention and that these expectations are based on their everyday lives [40]. Our study already includes interviews with participating patients with the purpose of examining patients’ expectations and experiences with the health dialogue. Though knowledge is still needed about how people understand, reason and act towards prevention in general practice in relation towards their everyday lives.

Our study draws upon ethnographic fieldwork and qualitative interviews from the process evaluation of the TOF pilot study, which means that all interviews and observations were performed in the context of the intervention framework and its embedded values towards health, risk and prevention. Generally, contextual circumstances such as risk perspectives, time, the health care system and physical arrangement are recognized as influencing the actions during and after preventive consultations [11, 44]. Thus, we found it relevant to consider in what way this affects the health dialogues. In this study, we find that the motivational work in the health dialogues was influenced by the digital support system, the GPs perceptions of their professional position as well as the patients’ assessment of health behavior as an individual affair. This means that the context of the intervention partly affected how motivational work was carried out in the health dialogues. Contrary to these findings, studies of preventive initiatives have showed that GPs’ prioritisation of trustful relationships with the patients could lead them to compromise or sidestep preventive health topics which hindered primary prevention in different ways [4, 8]. Thus, the influence of different kinds of contexts adds to the complexity of prevention in the clinical encounter and help to explain why motivational work is difficult in general practice.

Due to the evaluation design of the TOF pilot study, the focus of this study has been the preventive health dialogues. This means that attention has not been given to the preceding health examination. This means that the study does not include any views on motivational work carried out before the health dialogues.

## Conclusion

By exploring preventive health dialogues in general practice through the lens of motivational work, we found that different contextual factors relating to the intervention, the GPs understanding of professional authority and the patients understanding of prevention —in an interplay—influenced motivational work in the health dialogues to occur as one-way communication, characterized by biomedically based guidance, information, suggestions, and advice on risk factors. The findings point to some of the complexities and difficulties involved in implementing preventive initiatives as well as in providing preventive health advice in general practice in Denmark.

The findings, which point to the influence of different kinds of contexts, adds to the complexity of prevention in the clinical encounter which help to explain why motivational work is difficult in general practice.

## Supplementary information

**Additional file 1.** Interview guide questions for interviews with patients. Full set of questions from the interview guide with patients.

**Additional file 2.** Interview guide questions for interviews with GPs. Full set of questions from the interview guide with GPs.

## Data Availability

The sensitivity of the empirical material in this study entails that data sharing is not applicable.

## Abbreviations

GP: General Practitioner
TOF: ‘Tidlig Opsporing og Forebyggelse - a Danish acronym for ‘Early Detection and Prevention’
MI: Motivational Interviewing
