# Supplementary material for: ‘Motivational work’: a qualitative study of preventive health dialogues in general practice
Source: BMC Fam Pract. 2020 Sep 8;21:185. doi: 10.1186/s12875-020-01249-z (PMC7487907; doi:10.1186/s12875-020-01249-z)
Supplement: Supplementary file 1 — Additional file 1. Interview guide questions for interviews with patients. Full set of questions from the interview guide with patients. [file 12875_2020_1249_MOESM1_ESM.docx]

**Interview Guide - Patients**

**Formål:** *Borgernes oplevelser og vurdering af deres deltagelse i HUS-konceptet samt af invitation, spørgeskema og tilbagemelding i forbindelse med projektet*

**Aim:** The patients experience and assessment of their participation in the TOF pilot Study, as well as the invitation, the questionnaire and feedback in connection with the study.

1. Alder, beskæftigelse (hvad laver du til daglig?), civilstand.

Age, employment, civil status

1. Hvordan var det at være til Helbredsundersøgelse og Helbredssamtale ved din læge? Oplevede du, at din læge havde sat sig ind i din specifikke situation? Levede det op til dine forventninger? Hvorfor/hvorfor ikke?

How did you experience the health examination and the health dialogue at your GP? Did you find that your GP was familiar with your specific situation? Did it live up to your expectations? Why / why not?

1. Hvad fik dig til at tage imod tilbuddet? Hvilke tanker havde du gjort dig forud for helbredssamtalen?

Why did you accept the offer? What thoughts did you have prior to the health dialogue?

1. Har du tidligere overvejet selv at opsøge din læge for at få råd og vejledning ift. livsstil/sundhed og forebyggelse af livsstilssygdomme? Hvordan kan det være?

Have you previously considered consulting your GP for advice and guidance regarding lifestyle / health and prevention of lifestyle diseases? Why?

1. Hvordan var Helbredssamtalen anderledes end andre samtaler du har med din læge?

How was the health dialogue different from other consultations you have with your GP?

1. Havde du forberedt dig inden samtalen? Havde du udfyldt to skemaer inden konsultationen? Hvordan virkede det? Havde du forberedt dig på andre måder: fx snakket med nogen? Opsøgt information andre steder, fx hjemmesider, bøger mm.?

Did you prepare yourself on the health dialogue? Did you complete the two forms before the consultation? How did it work? Did you prepared yourself in other ways: e.g. talked to someone, seek out information elsewhere, e.g. websites, books etc.?

1. Hvad synes du om de forskellige spørgeskemaer du har skullet svare på? Hhv. TOF-spørgeskema (vurdering af din egen sundhed), Forberedelse til HUS-samtalen (vurdering af motivation og ressourcer) og balanceskemaet (fordele og ulemper ved adfærdsændring)

How did you like the questionnaires? Respectively the TOF questionnaire (assessment of your own health), Preparation for the health dialogue (assessment of motivation and resources) and the balance sheet (advantages and disadvantages of behavior change)

1. Hvordan har det været at bruge Sundhedsmappe.dk? Hvad synes du om invitationen og tilbagemeldingen du modtog over internettet/Sundhedsmappe.dk? Har du nogen forslag til forbedringer af sundhedsmappe.dk?

What is you experience of using the digital support system? What do you think about the invitation and feedback you received over the internet? Do you have any suggestions for improvements to the digital support system?

1. Hvordan synes du det var at få vurderet dit helbred? Har det sat nogle særlige tanker i gang?

What was it like to have your health assessed? Has it trigger any thoughts?

1. Hvad har du fået ud af Helbredsundersøgelsen og Helbredssamtalen – og hvordan vil du fremadrettet gøre brug af det? Hvad tænker du om det mål, du har sat sammen med din læge?

What have you gained from the health examination and the health dialogue - and how will you make use of it in the future? What do you think about the goals you have set with your GP?

1. Har du forslag til ændringer eller forbedringer ift. tilbuddet?

Do you have suggestions for changes or improvements in relation to the TOF intervention?
