# Supplementary material for: ‘Motivational work’: a qualitative study of preventive health dialogues in general practice
Source: BMC Fam Pract. 2020 Sep 8;21:185. doi: 10.1186/s12875-020-01249-z (PMC7487907; doi:10.1186/s12875-020-01249-z)
Supplement: Supplementary file 2 — Additional file 2. Interview guide questions for interviews with GPs. Full set of questions from the interview guide with GPs. [file 12875_2020_1249_MOESM2_ESM.docx]

# Interview guide – GPs

**Formål:** Lægernes oplevelser og vurdering af HUS-konceptet (helbredsundersøgelse + helbredssamtale) samt af det fælles orienteringsmøde.

**Aim:** The GP’s experiences and assessment of the TOF pilot study (health examination + health dialogue) as well as of the joint orientation meeting.

1. Alder, type af lægepraksis, hvor længe har du været praktiserende læge?

Age, type of medical practice, how long have you been a general practitioner?

1. Hvordan har I valgt at organisere og tilrettelægge opgaverne med TOF? Hvem i lægepraksissen varetager hvilke opgaver ift. helbredsundersøgelsen og bestilling af helbredssamtale? Hvordan fungerer det?

How did you organize the tasks with the TOF Pilot Study? Who in the medical practice handled which tasks in relation to the health examinations and booking of the health dialogues? How did it work?

1. Hvad er jeres motivation for at være med i projektet? Hvad er din opfattelse af lægens rolle som forebygger? (herunder evt. tilgange, metoder ift. forebyggelse)

What is your motivation for participating in the project? What is your perception of the GP’s role in preventing chronic disease? (including possible approaches, methods in relation to prevention)

1. Hvad er din umiddelbare vurdering af HUS-konceptets kvalitet og relevans ift. tidlig opsporing og forebyggelse?

What is your immediate assessment of the quality and relevance of the TOF pilot study in relation to early detection and prevention?

1. Hvordan er helbredssamtalerne anderledes end andre samtaler du har med patienter? Hvordan virker det fx, at du og borgeren har forberedt sig på en bestemt måde til samtalen? Hvordan fungerer det at skulle fastsætte et mål med borgeren og evt. henvise videre til kommunale tilbud, foreninger mm.?

How was the health dialogue different from other consultations? For example, how did it work that you and the patient were prepared for the dialogue? How did it work to set goals with the patient and the possibility to refer the patient to behavior change programs in the municipal?

1. Bruger du materialet fra TOF og den digitale platform før/under samtalen? Hvordan fungerer det? Oplever du oplysninger om borgeren fra TOF som brugbar for din samtale med borgeren? Hvorfor og hvordan? Hvorfor ikke?

How did you use the material from the TOF pilot study and the digital support system before/during the health dialogue? How did it work? Did you find the information about the patients useful in the health dialogue? Why and how? Why not?

1. Gør HUS-konceptet det lettere at snakke livsstilsændringer og forebyggelse med borgerne? Hvorfor/hvorfor ikke? Har du en fornemmelse af, om du når borgere, som du ikke tidligere har nået?

Does the material from the TOF pilot study makes it easier to talk about lifestyle changes and prevention with patients? Why? Why not? Do you experience that you are reaching patients you have not reach before?

1. Hvad tænker du om den måde borgerne bliver kategoriseret på (grøn, gul, rød, lilla)? Hvad kendetegner de ”røde” borgere? Hvordan oplever du, at HUS-konceptet virker på denne gruppe?

How do you experience the way patients are categorized (green, yellow, red, purple)? What characterizes the "red" group? How do you experience the TOF pilot study working on this group?

1. Hvordan fungerede det fælles orienteringsmøde i Kolding med læger og kommunale medarbejdere? Har du følt dig klædt på til at udføre HUS-konceptet? Hvad kunne have været anderledes?

What is your experience of the orientation meeting in Kolding with GPs and municipal employees? Have you felt prepared to carry out the TOF pilot study? What could have been different?

1. Oplever du, at det er lettere at henvise borgere til kommunale forebyggelsestilbud efter din deltagelse i TOF?

Is it easier to refer patients to behavior change programs in the municipal after your participation in the TOF Pilot study?

1. Hvad vil du vurdere jeres tidsforbrug ifm. HUS-konceptet til at være?

What is your assessment of the time you have spent on the TOF pilot study?

1. Hvordan tænker du, at HUS-konceptet kan forbedres?

How do you think the TOF pilot study can be improved?
